# Integrated Metagenomic and Metatranscriptomic Analyses of Microbial Communities in the Meso- and Bathypelagic Realm of North Pacific Ocean

**DOI:** 10.3390/md11103777

**Published:** 2013-10-11

**Authors:** Jieying Wu, Weimin Gao, Roger H. Johnson, Weiwen Zhang, Deirdre R. Meldrum

**Affiliations:** 1 Center for Biosignatures Discovery Automation, Biodesign Institute, Arizona State University, Tempe, AZ 85287-6501, USA; E-Mails: Jieying.Wu@asu.edu (J.W.); Weimin.Gao.2@asu.edu (W.G.); roger.h.johnson@asu.edu (R.H.J.); 2 School of Chemical Engineering and Technology, Tianjin University, Tianjin 300072, China

**Keywords:** deep-sea microbial community, metagenomics, metatranscriptomics, strength of metabolic activity

## Abstract

Although emerging evidence indicates that deep-sea water contains an untapped reservoir of high metabolic and genetic diversity, this realm has not been studied well compared with surface sea water. The study provided the first integrated meta-genomic and -transcriptomic analysis of the microbial communities in deep-sea water of North Pacific Ocean. DNA/RNA amplifications and simultaneous metagenomic and metatranscriptomic analyses were employed to discover information concerning deep-sea microbial communities from four different deep-sea sites ranging from the mesopelagic to pelagic ocean. Within the prokaryotic community, bacteria is absolutely dominant (~90%) over archaea in both metagenomic and metatranscriptomic data pools. The emergence of archaeal phyla *Crenarchaeota*, *Euryarchaeota*, *Thaumarchaeota*, bacterial phyla *Actinobacteria*, *Firmicutes*, sub-phyla *Betaproteobacteria*, *Deltaproteobacteria*, and *Gammaproteobacteria*, and the decrease of bacterial phyla *Bacteroidetes* and *Alphaproteobacteria* are the main composition changes of prokaryotic communities in the deep-sea water, when compared with the reference Global Ocean Sampling Expedition (GOS) surface water. Photosynthetic *Cyanobacteria* exist in all four metagenomic libraries and two metatranscriptomic libraries. In Eukaryota community, decreased abundance of fungi and algae in deep sea was observed. RNA/DNA ratio was employed as an index to show metabolic activity strength of microbes in deep sea. Functional analysis indicated that deep-sea microbes are leading a defensive lifestyle.

## 1. Introduction

The water body underlying the photic zone in the world’s oceans, representing the largest water mass on earth (comprising 1.3 × 10^18^ m^3^), is the largest aqueous habitat for microbial life [1]. This realm differs distinctly from the photic zone, presenting low temperature (approximately 2–4 °C), high pressure and high inorganic nutrient levels [2]. Differences in physical geochemical parameters between the upper level of sea water and deep sea suggest that microbial communities in these environments are confronted by fundamentally different challenges. Although emerging evidence indicates that deep-sea water contains an untapped reservoir of high metabolic and genetic diversity, and microbial communities in deep-sea water play an important role in ocean biogeochemistry [2,3,4,5]. Many marine microorganisms can synthesize various metabolic compounds. In recent decades (since the 1970s), a considerable amount of drug candidates were discovered from marine natural products [6]. These natural products are a rich source of new chemical diversity and also a vital component of the pharmaceutical industry [7]. Hence, the development of tools to access deep-sea microorganisms and microbial community promises to provide insight into this significant new source of drug discovery and development. Recent studies have found that prokaryotic plankton is one of the main drivers of biogeochemical cycles over large ocean expanses [8], and that eukaryotic microbes account for a significant fraction of the biomass and activity of marine microbial communities [9,10]. To better understand the influence of microbes on ocean geochemistry, we set about exploring the structure and metabolic characteristics of microbial communities in deep-sea environments. 

Progress in next-generation sequencing is fueling a rapid increase in the number and scope of microbial community-targeted studies [11,12,13,14,15,16]. While metagenomics provides information on the taxonomic composition and metabolic potential of a microbial community, metatranscriptomics serves to unveil the actual metabolic activities of the community at a specific time and place, and how those activities change in response to environmental forces or biotic interactions [17]. Defining the relationship between microbial community composition and function (metabolic characteristics) has been a major challenge in studying heterotrophic carbon cycling in marine systems [5]. The combination of metagenomic and metatranscriptomic approaches has proven efficacious in deciphering the phylogenetic composition, metabolic potential and pathways of deep-sea microbial communities. For example, in a recent study, coupled metagenomic and metatranscriptomic analyses were utilized for taxonomic and functional characterization of marine microbial communities living at depths between 25 and 500 m [18]. The results provided novel insight into not only microbial diversity but also specific metabolic processes transpiring in the ecosystems. Moreover, the different relative abundance of taxonomic groups identified in the metagenomic and metatranscriptomic libraries arising from the study revealed differential relative transcriptional activities per cell [18]. 

We present a study focused on simultaneous metagenomic and metatranscriptomic analysis of deep-sea microbial communities, including both prokaryotes and eukaryotes, from four different deep-sea sites ranging from the mesopelagic to pelagic ocean (depth of 784–1937 m, 2–4 m above the sea floor). Many marine microbial community studies have focused on either prokaryotes or eukaryotes [11,12,15,19,20,21]. To our knowledge, this is the first integrated metagenomic and metatranscriptomic study to include both prokaryotic and eukaryotic microorganisms living in the same habitat. DNA and RNA amplifications were performed to meet the entry requirements of nucleic acids for pyrosequencing. Integrated metagenomic and metatranscriptomic results revealed a defensive life style instead of active growing/metabolic style of both prokaryotic and eukaryotic communities living in the deep-sea water. Microbial community structures and their metabolic characteristics in the environments are presented and discussed. 

## 2. Results and Discussion

### 2.1. Overview of Data Generation and Analysis

Among the four sampling sites in this study, CT04, CT05, and CT06 are 164.2 km east of the coast, while CT12 is much further out to sea, approximately 20 km southwest of a deep-sea hydrothermal vent. Detailed information about the sampling sites is provided in Table 1. Although all sampling sites are close to the sea floor (2–3 m above the sea floor), sites CT05 and CT06 are in the mesopelagic realm (200–1000 m), while CT04 and CT12 are within the bathypelagic realm (1000–4000 m). By studying samples from four disparate sites we were better able to reveal a more complete picture of microbial communities’ structures and their metabolic characteristics in the deep-sea water of North Pacific Ocean.

**Table 1:** Sampling descriptions.

Sampling Sites	Position	Depth (m)	Sampling Time
CT04	44°29′34.80′′ N, 125°8′49.61″ W	1181–1194	7/23/2008, 21:00–21:24GMT
CT05	44°34′1.02″ N, 125°9′3.75″ W	785–790	7/27/2008, 3:05–7:21GMT
CT06	44°33′52.99″ N, 125°9′3.73″ W	763–789	7/27/2008, 3:12–7:31GMT
CT12	45°51′57.01″ N, 129°47′19.47″ W	1840–1913	8/2/2008, 00:01–00:24GMT

In natural environments such as the deep sea, microbial cell density can be as low as 10^3^–10^4^ cells mL^−1^ of seawater [22]. In addition, RNA abundance decreases with depth due to a relatively slow metabolism [23]. Compared to biomass-abundant samples such as surface seawater, the intact RNA that can be isolated from these environments is very limited in quantity: usually low to several hundred nanogram level. On the other hand, typical commercial next-generation sequencing platforms require 3–5 μg or more of input DNA/cDNA to produce reliable sequencing data, approximately equal to the total amount of DNA isolable from 10^9^ *Escherichia coli* cells [24]. Due to the limited samples collected from the deep-sea water, an amplification process was employed in this study for both genomic DNA and total RNA samples to provide sufficient DNA and cDNA for metagenomic and metatranscriptomic analysis. Total RNA was used for metatranscriptomic analysis, allowing simultaneous assessment of rRNA and mRNA to produce both taxonomic and metabolic information on the studied microbial communities [18,25]. 

Using the amplification method [24], we generated 4.9–16 μg of the final products from 5.0–20.0 ng of DNA/RNA template, with only ~600 ng DNA detected in negative controls. The statistical summary of the sequenced data determined by MG-RAST is shown in Table 2. The Nugen Ovation WGA system (NuGEN, San Carlos, CA) used for DNA amplification has been evaluated by the manufacturer and demonstrated the ability to faithfully replicate genomic DNA. The performance of WT-Ovation™ Pico RNA Amplification System (NuGEN) used for total RNA amplification in this study has been evaluated in two other studies and shown to be reproducible with minimal bias [26,27]. Pyrosequencing of community DNA and RNA across four deep-sea sampling sites generated 160,072 and 64,928 sequencing reads (after quality control and de-replication), with mean lengths of 225.5 bp and 182.2 bp, respectively. Table 3 shows the microbial community compositions of different samples as revealed by metagenomic and metatranscriptomic analysis. The results demonstrate that the taxonomic compositions of microbial communities as revealed by metagenomic analysis differ markedly from those obtained by metatranscriptomic analysis, especially for archaeal and bacterial groups, suggesting the importance of using both approaches to avoid possible methodological bias. In metagenomic and metatranscriptomic data, the proportion of eukaryotic reads was much higher than that of archaeal and bacterial reads (Table 3). The proportion of eukaryotic reads (58.73%–71.92% based on metagenomic analysis, and 73.45%–87.54% based on metatranscriptomic analysis) in this study without pre-filtration were almost an order of magnitude higher than those in several previous studies using pre-filtered sea water, which were typically under 5% [19,28,29], but was similar to one recent study which reported higher proportions of eukaryotes at different depths (10 m, 25.60%; 800 m, 48.08%; 4400 m, 37.27%) in the North Pacific Ocean [30]. 

**Table 2:** Statistical summary of the sequencing results.

	DNA				RNA			
	CT04	CT05	CT06	CT12	CT04	CT05	CT06	CT12
Total reads	61,650	46,012	50,491	40,049	12,804	13,722	18,367	33,509
Ave. length (bp)	217 ± 143	228 ± 145	252 ± 156	236 ± 144	170 ± 113	184 ± 120	177 ± 124	202 ± 121
Ave. GC%	49 ± 8	50 ± 8	48 ± 8	48 ± 8	49 ± 6	36 ± 6	49 ± 7	48 ± 6
Failed QC	15,938	11,537	11,133	0	3745	4421	5443	0
Annotated protein	8545	7328	9070	12,720	201	0	0	10,300
Unknown protein	18,581	14,359	17,011	15,723	4602	5790	6981	13,569
Ribosomal RNA	1505	1042	999	2811	1932	1009	3663	6175
Unknown	17,081	11,746	12,278	8795	2324	2502	2280	3465

**Table 3:** Taxonomic compositions of metagenomic and metatranscriptomic libraries.

	Metagenome (%)				Metatranscriptome (%)			
	CT04	CT05	CT06	CT12	CT04	CT05	CT06	CT12
Archaea	1.31	1.97	4.23	1.60	1.24	0.80	0.64	1.05
Bacteria	26.56	30.27	34.30	24.16	23.06	9.42	17.14	19.98
Eukaryotes	69.94	65.73	58.73	71.92	73.45	87.54	81.40	78.37
Viruses	0.41	0.29	0.47	0.44	0.30	0.50	0.23	0.25
Others *	1.78	1.74	2.26	1.88	1.96	1.77	0.59	0.35

* Others: unclassified sequences.

To better define the characteristics of deep-sea microbial communities, we also employed, as a reference for the data analysis, metagenomic data from the microbial community isolated from surface waters (sampling depth: 1 m) of Browns Bank, Gulf of Maine, in the Global Ocean Sampling Expedition (GOS) project [29]. We chose this data set as our reference in part because the timing of the GOS project’s sample collection was similar to that in our study (GOS: August 21th, 2003); and in part because its sampling location, especially the latitude, was also relatively close to that of our study (GOS: +43°39′53.95′′, −65°33′50.78′′). 

### 2.2. Metagenomic Analysis of the Deep-Sea Prokaryotic Communities

**Figure 1:**
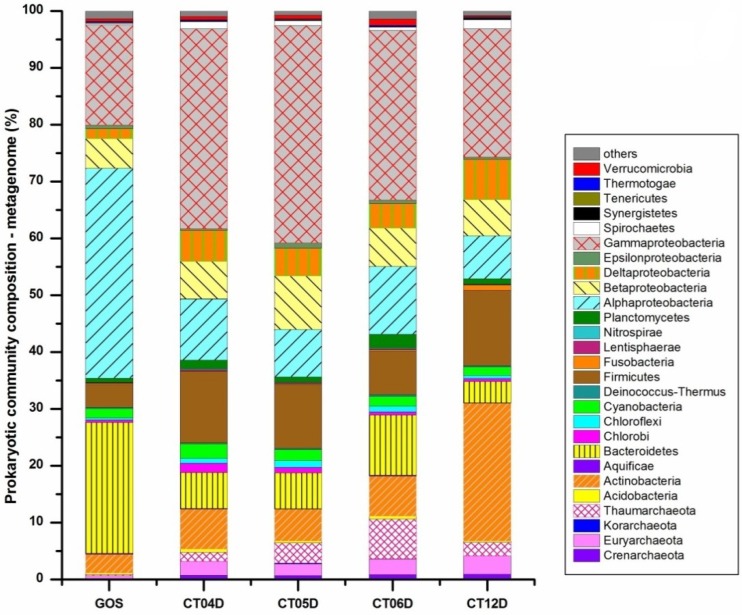
Comparison of prokaryotic microbial communities’ composition in four deep-sea sampling sites and in Global Ocean Sampling Expedition (GOS) surface water.

When compared with archaea, bacterial reads represented more than 90% of the prokaryotic sequencing reads from both metagenomic and metatranscriptomic data for all sampling sites, suggesting that bacteria are absolutely dominant in the prokaryotic communities in the deep-sea water samples. This is similar to previous studies conducted on soil, surface water, deep sea, and marine sediment [25,28,29,30,31]. However, archaea were typically found at higher levels in the marine sediment than in sea water [32]. Figure 1 shows the compositions of prokaryotic communities in metagenomic and metatranscriptomic data from four sampling sites and the reference surface water community revealed by metagenomic data [29]. The results revealed remarkably high microbial diversity even though the cell densities were much lower in the deep sea. Prokaryotic communities of the GOS surface water and the four deep-sea sampling sites diverged significantly in terms of phylogenetic composition at broad levels of phyla and classes (Figure 1). In the surface water, the proportion of archaea was less than 1% of the prokaryotic community, while in the deep sea, it was increased to as high as 13.54% (CT06). This result is in accord with a general trend observed in multiple ocean basins: the proportion of archaea increases with depth [33,34]. The changes from GOS surface water to deep-sea water were primarily manifest by the emergence of archaeal phyla *Crenarchaeota*, *Euryarchaeota* and *Thaumarchaeota*, bacterial phyla *Actinobacteria* and *Firmicutes*, sub-phyla *Betaproteobacteria*, *Deltaproteobacteria* and *Gammaproteobacteria*, and the decreasing abundances of bacterial phyla *Bacteroidetes* and *Alphaproteobacteria*. Among them, phylogenetic lineages within chemolithoautotrophic *Thaumarchaeota*, *Betaproteobacteria* and *Gammaproteobacteria* were recognized as ammonia oxidizing archaea (AOA) and ammonia oxidizing bacteria (AOB) that can oxidize ammonia to nitrite [35]. This process is the first and rate-limiting step in nitrification and is also a vital component of the global biogeochemical nitrogen cycle [36]. The AOB *Betaproteobacteria* and *Gammaproteobacteria* are considered a major mediator of ammonia oxidation processes [37]. Previous study has confirmed that at least some AOA (e.g., *Nitrosopumilus*, one of the main members identified in the *Thaumarchaeota* group in this study) have a high ammonia affinity and can grow in extremely oligotrophic environments [38]. Considering the trace substrate concentration in the deep sea, AOA *Thaumarchaeota* may be the major nitrifier in that environment [39]. Figure 1 also reveals a slight increase of *Planctomycetes* in the deep sea, all of which are members of the well-known anammox bacterial genus identified previously in the marine sub-oxic zone including *Rhodopirellula*, *Blastopirellula*, *Planctomyces*, *Pirellula*, *Candidatus Kuenenia*, *Gemmata* and *Isosphaera* [40,41]. The presence of *Planctomycetales* species *Candidatus Brocadia* and *Candidatus Kuenenia* in the low-oxygen, dark pelagic ocean is an indicator that anaerobic ammonium oxidation (anammox) [42], a globally important microbial process of the nitrogen cycle, may be another metabolic pathway supporting primary production in this environment [43]. More information on environmental parameters, especially oxygen concentration, will be required before a definitive conclusion can be reached. *Actinobacteria* and *Firmicutes* had higher representation in the deep-sea prokaryotic community compared to surface water, probably due to the adaptive advantage of *Actinobacteria* and *Firmicutes* under low-nutrient conditions of the deep sea [44]. Moreover, it has been proposed that the ability of *Actinobacteria* to survive in cold and dystrophic environments might be due to its adaptive ability to go into resting states with low metabolic activity [45]. 

Our results demonstrated a dramatic decrease of *Alphaproteobacteria* in the deep sea (Figure 1), consistent with a previous study at the Hawaii Ocean Time-series (HOT) station ALOHA [28]. In the *Alphaproteobacteria* group, *Candidatus pelagibacter*, an abundant member of the SAR11 clade [46] and one of the most abundant groups of bacteria in the upper surface waters of the oceans [29,47], was found to be the most conspicuously decreased genus in the deep-sea samples. The SAR11 clade was found to contribute to the variability in utilization of nutritional compounds (glucose, ATP, a combination of amino acids, and organic compounds, the sources of C, N, and P) by the bacterial community, and its activity can be linked to the bacterial community’s activity as a whole because of its ability to adapt to nutrient limitation [48]. Hence, the decreased representation of *Candidatus* Pelagibacter in the deep-sea prokaryotic communities indicated lower levels of nutrient utilization and bacterial community activity in the deep sea relative to surface water. In the surface water, class *Flavobacteria* dominated within the *Bacteroidetes* phylum, while in the deep sea the taxonomic composition within *Bacteroidetes* changed to a mixed assemblage of *Bacteroidia* and *Prevotella*. This represented a shift from aerobic to anaerobic phylotype, that is, aerobic *Flavobacteria* in surface waters giving way to most anaerobic *Bacteroidia* and *Prebotella* spp in the deep-sea water, consistent with the decrease in dissolved oxygen (DO) in deep sea relative to surface environments.

### 2.3. Metatranscriptomic Analysis of the Deep-Sea Prokaryotic Communities

**Figure 2:**
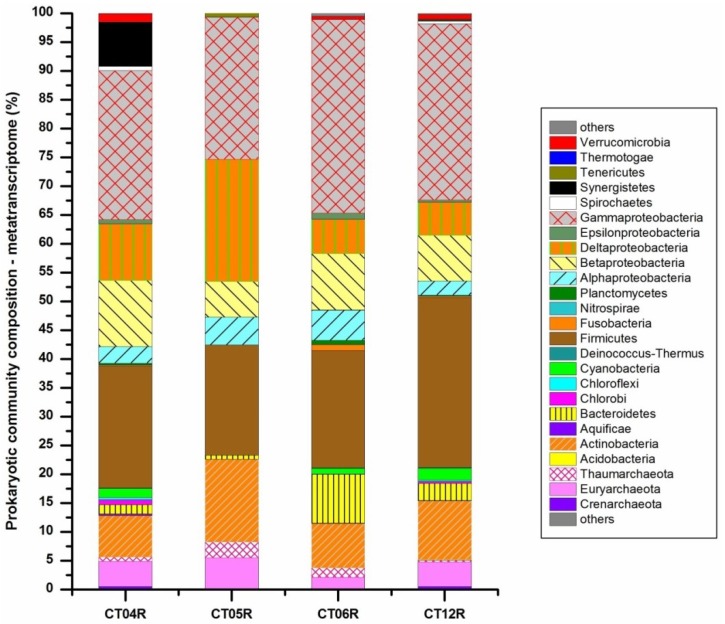
Metabolically active prokaryotes in deep sea.

Of the 38 total prokaryotic phyla identified in metagenomic libraries from the four samples, there were 20 phyla in CT04, 10 phyla in CT05, 17 phyla in CT06, and 26 phyla in CT12 also detected in the metatranscriptomic libraries (Figure 2). Of the four deep-sea sampling sites, CT12 harbored the most diverse metabolically active prokaryotes. In the metatranscriptomic libraries of all four samples, *Gammaproteobacteria* constituted the highest proportion, similar to the metagenomic libraries. Compared to prokaryotic community composition as explored from metagenomic data, proportions of *Crenarchaeota*, *Thaumarchaeota*, *Alphaproteobacteria*, *Bacteroidetes* and *Planctomycetes* were decreased markedly, while *Euryarchaeota*, *Deltaproteobacteria*, *Firmicutes* and *Actinobacteria* were increased in the metatrascriptome libraries (Figure 2). The archaeal phyla *Korarchaeota* and *Nanoarchaeota*, and bacterial phyla *Poribacateria*, *Chrysiogenetes*, *Deferribacteres*, *Elusimicrobia*, *Fibrobacteres*, *Gemmatimonadetes*, *Lentisphaerae* and *Zetaproteobacteria*, which made up a minor portion of the metagenomic libraries, were completely absent in the metatranscriptome libraries from all four deep-sea sites. As discussed in more detail below, the relative abundances of these taxonomic groups (phylum level) were very different in the metagenomic and metatranscriptomic libraries, indicating differential relative transcriptional activities per cell [18]. 

### 2.4. Integrated Metagenomic and Metatranscriptomic Analysis of Prokaryotic Communities in Deep-Sea Water

**Figure 3:**
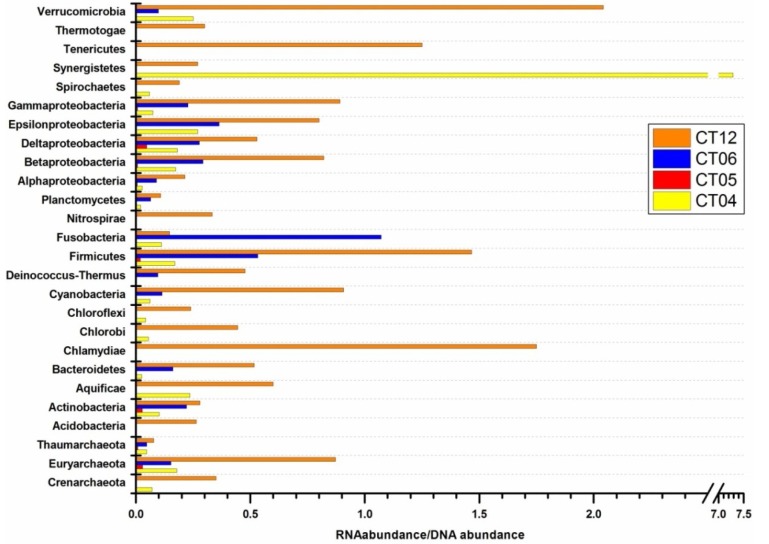
Comparison of metabolic activity strength using RNA/DNA ratios of prokaryotic communities in deep sea.

From the metagenomic analysis, a total of 453, 462, 598 and 564 different prokaryotic genera were identified for site CT04, CT05, CT06 and CT12, respectively; while from the metatranscriptome analysis, the numbers of detected genera were 113, 34, 138 and 332. The number of species identified and overall community diversity revealed by metatranscriptomic data was relatively low compared to the metagenomic data. Metagenomic analysis provides better coverage of microbial species but lacks the information afforded by metatranscriptomic analysis on the metabolic activities of the communities described. Given the differences observed between metagenomic and metatranscriptomic analysis, it is clear that neither metagenome-based nor metatranscriptome-based analysis is sufficiently comprehensive to fully characterize a microbial community. In this study we defined a parameter to measure the strength of metabolic activity at the genus level as the ratio of transcript abundance in the RNA pool to gene abundance in the DNA pool. The abundances calculated and provided by MG-RAST are counts of taxon (the abundance represents the number of times a particular taxon is detected) or function (each count represents the number of times a particular functional role is detected) [49]. Prokaryotic genus with very low abundance values (as low as 1), or lack of either DNA or RNA data, was excluded from the analysis. Figure 3 shows the RNA/DNA ratio of the major prokaryotic classes identified in the deep-sea samples. We found that the RNA/DNA ratio of single phylum/sub-phylum varied considerably among the deep-sea sites, likely reflective of the different metabolic conditions the prokaryotic communities were experiencing in the varied environments (Figure 3). Overall, the prokaryotic community in site CT12 (20.0 km away from a hydrothermal vent on the Juan de Fuca Ridge [50]) showed the highest RNA/DNA ratios among the four sites for almost all detected phyla except Synergistetes, consistent with the relatively higher temperature and mineral-rich environment which support highly diverse and more active communities of microbes around hydrothermal vents [51,52]. Due to the small taxonomic coverage from the RNA pool, RNA/DNA ratios were missing for most of these phyla in CT05, except for phyla Euryarchaeota, Thaumarchaeota, Actionbacteria, Firmicutes and Proteobacteria.

**Figure 4:**
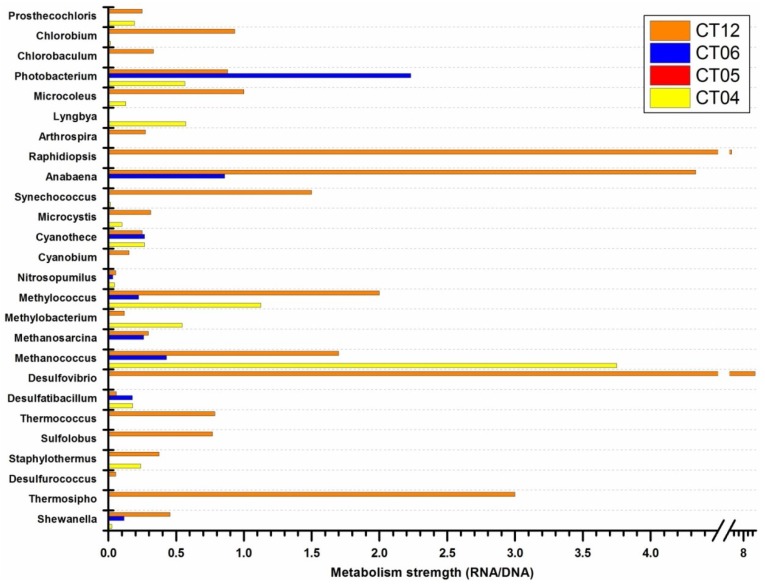
Metabolic activity strength comparison using RNA/DNA ratios of selected prokaryotic genera.

Figure 4 shows the RNA/DNA ratios of some interesting prokaryotic genera identified in the studied samples. Here RNA/DNA ratio was employed as an index to show strength of metabolic activity (SMA) of prokaryotes in deep-sea environments. Piezophilic bacteria *Shewanella*, thermophilic archaea/bacteria, sulfur/sulfate reducing bacteria, methanotrophic archaea/bacteria, genera within the green sulfur bacteria *Chlorobia* group, and *Cyanobacteria* genera detected in the deep-sea samples were chosen as targets. Of all the sites, overall, deep-sea site CT12 harbored the largest number of genera showing high potential SMA (Figure 4), even for the photoautotrophic *Cyanobacteria* group. Considering the metabolic characteristics of these targeted genera (piezophilic, thermophilic, methanotrophic and sulfur/sulfate reducing), along with the unique physico-chemical properties of hydrothermal vents or proximal sites [20], we infer that though CT12 is 20 km away from the hydrothermal vent, it is still under its environmental influence.

**Figure 5:**
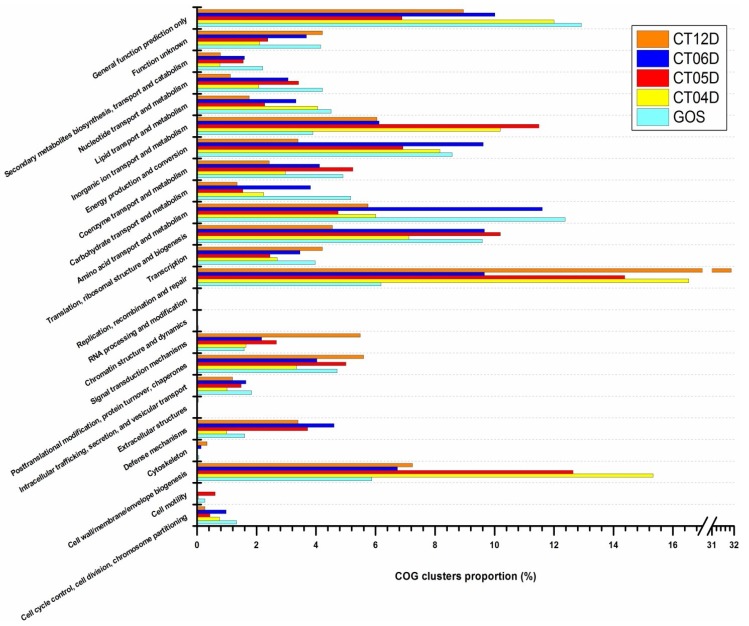
Clusters of Orthologous Groups (COG) distributions of prokaryotic metagenomic libraries.

To functionally annotate the sequences collected from each site, the metagenome and metatranscriptome reads obtained were searched against Clusters of Orthologous Groups (COG) of the National Center for Biotechnology Information (NCBI). The COG function clusters revealed in the DNA and RNA data showed huge differences in their diversity and the number of reads assigned to each category (Table 3). Unique functions identified in metatranscriptomic data were less diverse than those in the metagenomic data, probably due to the fact that only a fraction of prokaryotic organisms were metabolically active and expressing their functional genes or because, even though many genes were expressed, their expression levels were lower than the built-in detection threshold of MG-RAST (Table 3). Assuming the density of sea water to be 1025 kg/m^3^, pressure increases by 1 atm with each 10 m of depth (www.calctool.org). Based on this premise, we estimated that the hydrostatic pressure in our sampling sites ranged from 77.29 to 190.78 atm, with the highest pressure present at the deepest site CT12. Hence, in contrast to the microbial communities near the surface, genes involved in COG clusters detected in deep-sea metagenomic libraries showed characteristics strongly associated with the high hydrostatic pressures including “cell wall/membrane/envelope biogenesis”, “cytoskeleton”, “defense mechanisms”, “signal transduction mechanisms”, “replication, recombination and repair”, and “inorganic ion transport and metabolism” (Figure 5). The transcriptional potential of protein-coding genes varied remarkably among the sampling sites. In particular, genes from GOS surface water had higher representation in COG clusters including “cell cycle control, cell division, chromosome partitioning”, “amino acid transport and metabolism”, “carbohydrate transport and metabolism”, “lipid transport metabolism”, “nucleotide transport and metabolism”, “secondary metabolites biosynthesis”, and “transport and catabolism” (Figure 5). 

#### 2.4.1. Cell Wall/Membrane/Envelope Biogenesis

Several functional groups related to lipopolysaccharide biosynthesis were found, including LPS: glycosyltransferases (COG1442), Dihydrodipicolinate synthase/N-acetylneuraminate lyase (COG0329), Glycosyltransferases involved in cell wall biogenesis (COG0463), and a predicted sugar phosphate isomerase involved in capsule formation (COG0794). Lipopolysaccharide (LPS) constitutes the outermost leaflet of the outer membrane of gram-negative bacteria [53], while dihydrodipicolinate synthase (DHDPS) catalyses the first step in the biosynthetic pathway producing meso-diaminopimelate (DAP) and (S)-lysine, required components of the cell wall [54]. The higher proportion of “Cell wall/membrane/envelope biogenesis” related genes in deep-sea samples compared to surface water is probably due to the requirement for cell wall integrity under high pressure [18].

#### 2.4.2. Signal Transduction Mechanisms

COG cluster “signal transduction mechanisms” including Signal transduction histidine kinase (COG 0642), FOG: PAS/PAC domain (COG 2202), FOG: CheY-like receiver (COG0784), a predicted membrane GTPase involved in stress response (COG1217), and FOG: GGDEF, and GAF domain (COG2199, COG 2203) were identified in the metagenomic data of deep-sea prokaryotic communities. Signal transduction histidine kinase (COG 0642) and FOG: PAS/PAC domains (COG 2202) are two groups that were particularly enriched in deep-sea protein coding genes. Genes encoding histidine kinase are important for chemotaxis and quorum sensing [55]. The PAS domain is integral to proteins that sense environmental stimuli such as oxygen and redox potential [56]. The proportion of signal transduction pathways evident in deep-sea prokaryotic communities was higher than in GOS surface water, a manifestation of the need for deep-sea prokaryotic communities to sense and adapt to dystrophic deep sea environments, and consistent with previous findings from a study on microbial communities at 6,000 m depth in the Puerto Rico Trench [21]. 

#### 2.4.3. Replication, Recombination, and Repair

In this category, Recombinational DNA repair protein (*RecF* pathway) (COG0353) and Adenine specific DNA methylase Mod (COG2189) are major functions among the functions array detected in the metagenomic data from the deep-sea water samples. Methylation of DNA by the DNA adenine methylase plays an important role in DNA mismatch repair and replication regulation [57]. The enrichment of DNA repair protein coding genes probably indicated the strong DNA repair capacity of prokaryotes in deep-sea water to protect themselves from DNA degradation in the harsh environments [58], increasing their likelihood of survival [28].

#### 2.4.4. Inorganic ion Transport and Metabolism

Outer membrane receptor proteins, mostly Fe transport (COG1629), is the most dominant function detected in this category for all four deep-sea sampling sites. Iron is an important component of most redox enzymes [59]. We also detected the sequences of functional genes associated with membrane transport of heavy metals such as Co, Cu, Zn, Mn and Cd in site CT12. It has been reported that some microorganisms in hydrothermally active areas can remove heavy metals from hydrothermal fluid [60]. The presence of these functional genes supported our hypothesis that the microbial community in CT12 was under the influence of the hydrothermal vent 20 km away. 

In metatranscriptomic libraries of deep-sea prokaryotic communities, far more limited COG clusters were identified compared to the metagenomic libraries. The transcriptional activity of protein-coding genes varied distinctly among the four sampling sites. In total 24 functional categories were identified in the metagenomic libraries; 6 in CT04, 9 in CT06 and 15 categories in CT12 were characterized (Table 4). Only two COG groups, Transcriptional regulator (COG0583) and Ribosomal protein S3 (COG0092) were identified in CT05. Under the cell wall/membrane/envelope biogenesis category, the two bathypelagic sites CT04 and CT12 showed significantly higher percentages than the other two sites. In the metagenomic pool, this category accounted for 15.33% in CT04 and 7.24% in CT12; while in the metatranscriptomic pool, the percentages were increased to 60.87 and 40.53, respectively. While the increase may be due to the smaller number of COG categories detected in the metatranscriptomic pool, it still shows that mRNA related to cell wall/membrane/envelope biogenesis was more highly expressed in the two deeper sites compared to the shallower CT05 and CT06 sites. “Replication, recombination and repair” was a category occupying a high percentage in all deep-sea sites except CT05. Serine/threonine protein kinase (COG0515) and Adenine specific DNA methylase Mod (COG2189) enriched this category. Sites CT12 and CT06 showed relatively higher percentages of the category “Inorganic ion transport and metabolism”, which was enriched by Outer membrane receptor proteins (COG1629) in CT06, and Outer membrane receptor proteins (COG1629) and ABC-type phosphate/phosphonate transport system ATPase component (COG3638) in CT12. Hence, the defensive life style of prokaryotes in the deep sea could be inferred from metatranscriptomic analysis [19,61] since the detected mRNA COG categories were mostly related to cell wall/membrane and capsule formation for high pressure resistance, nucleotide repair, and membrane transporter for virulence.

**Table 4:** Prokaryotic protein coding genes assignment to COG in metagenomic and metatranscriptomic libraries.

	Metagenomics				Metatranscriptomics			
	CT04	CT05	CT06	CT12	CT04	CT05	CT06	CT12
**Unique COG Functions Identified**	401	406	876	528	8	2	20	85
**Sequences Assigned to COG Functions**	5971	5745	2336	2841	166	6	306	877

### 2.5. Active Presence of Photosynthetic Bacteria in Deep-Sea Water

Photoautotrophic microbes such as genera *Prochlorococcus* and *Synechococcus* are abundant members of the microbial community in the euphotic zone and responsible for much of the primary production in the ocean [62,63]. Meanwhile, early studies have suggested that viable photoautotrophic microbes such as *Synechococcus* sp. can be found in deep sea water or sediments exposed to little or no sunlight [19,64]. However, these studies provided no evidence as to whether these photoautotrophic microbes were metabolically active in the deep sea, or merely present as dormant cells. In our previous study, in addition to direct auto-fluorescence imaging, the presence of photoautotrophic microbes in the deep mesopelagic zone (765–790 m) was demonstrated by both bacterial 16S and 23S rRNA-based clone library analysis, and the metabolic activity of the oxygenic photoautotrophic bacteria *Cyanobacteria Synechococcus* sp. was demonstrated by expression level quantification of the 23S rRNA gene involved in protein synthesis and photosynthesis-involved gene *psbA* using RT-qPCR [65]. Here we employed metagenomic and metatranscriptomic analysis to further demonstrate the existence of active photosynthetic microbes in samples from four deep-sea sites. Photoautotrophic bacterial phylum *Cyanobacteria* (including genera *Cyanobium*, *Cyanothece*, *Synechococcus*, *Anabaena*, *Cylindrospermopsis*, *Nodularia*, *Nostoc*, *Arthrospira*, *Lyngbya*, *Microcoleus* and *Oscillatoria*) were found by both metagenomic and metatranscriptomic analysis in samples from all four sites, further suggesting the presence of metabolically active photosynthetic bacteria in the pelagic ocean realm. SEED subsystems identified in both metagenomic and metatranscriptomic pools are listed in Supplemental Table S1. In contrast to the diverse *Cyanobacteria* subsystems identified in metagenomic data, only a few were identified from the metatranscriptomic data. This might be due to a combination of the low quality of the RNA samples and the low metabolic activity of deep-sea *Cyanobacteria*. In site CT05, no *Cyanobacteria* SEED subsystems were detected. The Photosystem II protein D1 encoding gene (*psbA*) was detected in the metatranscriptomic pool of CT06, which belongs to *Nostoc* sp. (strain PCC 7120/UTEX 2576), suggesting that a “photosynthesis” pathway may be still maintained when *Cyanobacteria* find themselves in deep-sea environments. Counterintuitively, the most diverse *Cyanobacteria* subsystems were identified in samples from the deepest site, CT12, the site also most close to the hydrothermal vent.

The reason for the presence of metabolically active *Cyanobacteria* may be the sinking mechanism of marine particles. Cell aggregation phenomena are frequently observed in many *Cyanobacteria* species [66]. A study on particle interceptor traps at the Bermuda Atlantic Time-series Study found that the *Cyanobacteria* *Prochlorococcus* and *Synechococcus* were consistently detected in the water column, and that they trap samples at different depths in the euphotic zone, showing that *Cyanobacteria* can contribute to downward particle flux [67]. Further study is necessary to provide more information about the active presence of *Cyanobacteria* in dark, deep-sea environments.

### 2.6. Taxonomic and Functional Study of the Deep-Sea Eukaryotic Community

**Figure 6:**
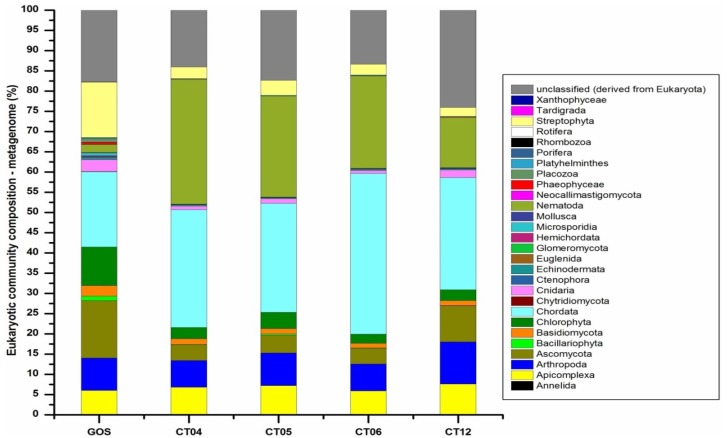
Eukaryotic community compositions in deep sea.

In line with our observations regarding the deep-sea prokaryotes, deep-sea eukaryotic communities displayed remarkably different compositions from those in the GOS surface water (Figure 6). Though eukaryotes accounted for a far higher proportion in the sampled deep-sea microbial communities (58.73%–71.92% in metagenomic data) than in the GOS surface water (3.31% in metagenomic data) [29], the diversity of the deep-sea eukaryotic community was very limited: In the GOS surface water, 25 different eukaryotic phyla were identified, while 17, 17, 18 and 20 eukaryotic phyla were identified at the deep-sea sampling sites CT04, CT05, CT06 and CT12, respectively. In contrast, fungi *Chytridiomycota*, flagellate *Euglenida*, parasite *Rhombozoa*, animal *Tardigrada*, and yellow-green algae *Xanthophyceae* were absent in all four deep-sea sites. Besides, the community changes from the GOS surface water to the deep-sea water were primarily manifest in the emergence of phyla *Chordata* and *Nematoda*, and the decrease of fungi *Ascomycota*, *Basidiomycota*, green algae *Chlorophyta*, plant *Streptophyta* and *Cnidaria*. Fungus *Chytridiomycota*, *Glomeromycota*, *Microsporidia*, *Neocallimastigomycota*, algae *Phaeophyceae*, *Xanthophyceae*, diatom *Bacillariophyta* and other animal phyla were also identified in the deep-sea metagenomic data, although making up only a small proportion of the total. Because we studied the microbial diversity using non-size-fractionated deep-sea samples, animalia *Chordata* and *Nematode*, whose sizes are typically around 2.50 mm, turned out to be dominant groups here. The proportion and diversity of fungi was also found to be decreased in the deep-sea water. In the GOS surface water, 100 fungus species were identified; while in the deep-sea water 63, 66, 68 and 75 different fungus species were identified in sites CT04, CT05, CT06 and CT12, respectively. The decrease in fungus was also reported in a previous study which reported that, compared to surface water, fungi were rare and less diverse in high-pressure, deep-sea environments [68]. Our results also identified three phyla belonging to eukaryotic parasites, including *Apicomplexa*, *Annelida* and *Nematode*. *Apicomplexa* were commonly found in deep-sea environments including hydrothermal vents [69] and methane cold seeps [70]. *Annelida*, which we identified with very small abundance in site CT12, is one of the few parasites found in hydrothermal vents [71]. Another detected eukaryotic parasite, *Nematodes,* is among the most abundant metazoan taxa in deep-sea ecosystems in general [72,73,74].

**Figure 7:**
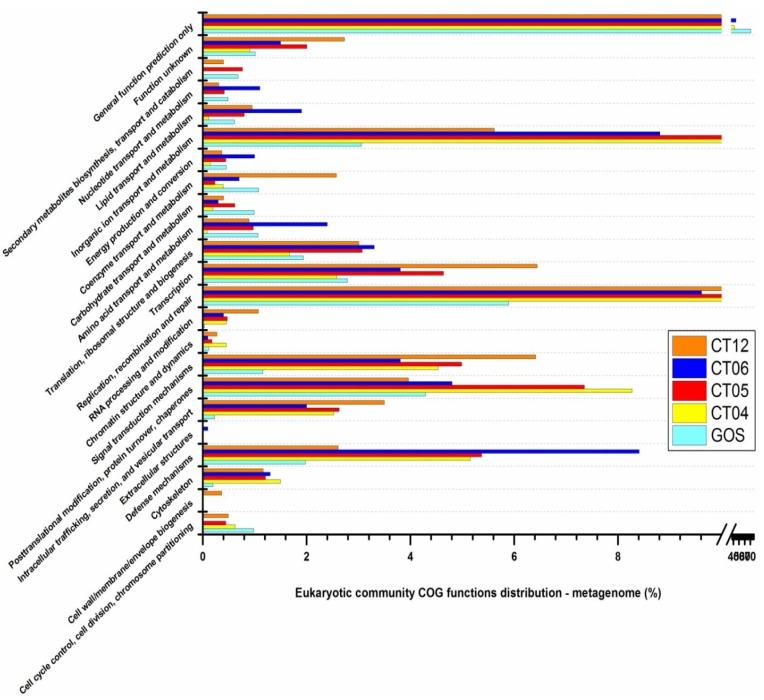
COG distributions of eukaryotic metagenomic libraries.

**Figure 8:**
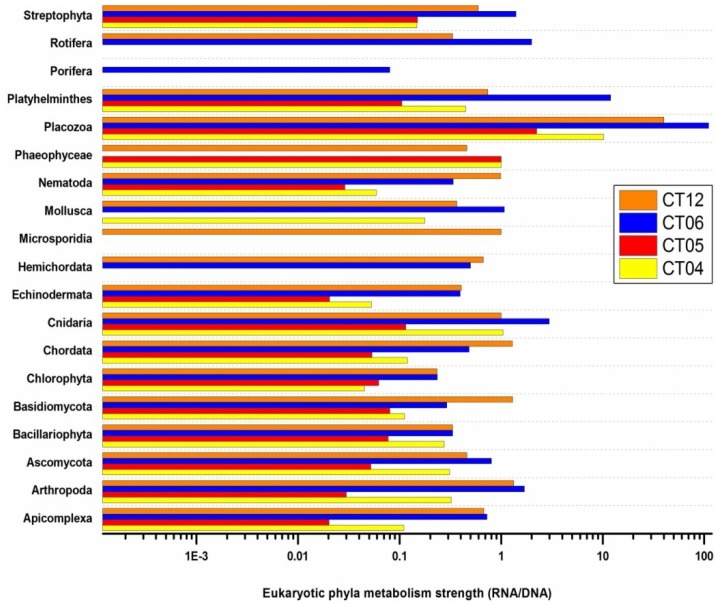
Metabolic activity strength comparison using RNA/DNA ratios of eukaryotic communities in deep sea.

Searching against the COG database, we found a similar trend in functional categories for deep-sea eukaryotes and prokaryotes in the metagenomic libraries (Figure 7). In contrast to GOS surface water, deep-sea eukaryotic protein coding genes are more involved in functions such as “defense mechanisms”, “intracellular trafficking, secretion, and vesicular transport”, “signal transduction mechanisms”, “replication, recombination and repair” and “inorganic ion transport and metabolism”, which are useful for leading a defensive life style as discussed in the section on prokaryotes. For deep-sea eukaryotic communities relatively limited COG clusters were identified in the metatranscriptomic libraries compared to the metagenomic libraries. The transcriptional activity of protein-coding genes varied remarkably among the four sampling sites, and it was difficult to identify any obvious trends analogous to those identified in the metagenomic libraries (Figure 8). Of the 23 functional categories identified in the metagenomic libraries, 17 categories in CT04, 8 in CT05, 15 in CT06 and 20 categories in CT12 were identified in their metatranscriptomic pool: better coverage compared to the prokaryotic metatranscriptomic libraries, possibly due to the larger number of sequencing reads obtained (Table 5). Among the four deep-sea metatranscriptomic libraries, site CT12 showed the highest percentage of genes related to the functional category “inorganic ion transport and metabolism”. This category from CT12 was mainly enriched with clusters including “Outer membrane receptor proteins, mostly Fe transport” COG0629, “Predicted divalent heavy-metal cations transporter” COG0428, and “Cation transport ATPase” COG0474. For the categories “signal transduction mechanisms” and “replication, recombination and repair”, site CT12 also harbored a significantly higher percentage of genes than other three sites. “Diadenosine tetraphosphatase and related serine/threonine protein phosphatases” COG0639 and “Universal stress protein UspA and related nucleotide-binding proteins” COG0589 are two main components included in this category. Site CT05 showed discernibly lower metabolic strength in most eukaryotic phyla, while CT06, very close and at similar depth to CT05, showed the highest metabolic strength in almost all eukaryotic phyla, similar to our findings in the deep-sea prokaryotic study. 

**Table 5:** COG clusters identified in metatranscriptomic libraries of deep-sea prokaryotic communities

Level 1	Level 2	CT04	CT05	CT06	CT12
*Cellular processes and signaling*	Cell cycle control, cell division, chromosome partitioning	0	0	0	0.12
	Cell wall/membrane/envelope biogenesis	60.87	0	19.46	40.53
	Defense mechanisms	0	0	0	0.12
	Intracellular trafficking, secretion, and vesicular transport	0	0	0	0.23
	Posttranslational modification, protein turnover, chaperones	12.17	0	4.70	1.51
	Signal transduction mechanisms	0	0	0	1.51
*Information storage and processing*	Replication, recombination and repair	21.74	0	27.52	26.13
	Transcription	0	33.33	14.09	3.83
	Translation, ribosomal structure and biogenesis	0	66.67	0	0.35
*Metabolism*	Amino acid transport and metabolism	0.87	0	4.70	0.58
	Carbohydrate transport and metabolism	0	0	0	0.46
	Energy production and conversion	0.87	0	7.38	0
	Inorganic ion transport and metabolism	0	0	9.06	12.89
	Lipid transport and metabolism	0	0	0	0.12
	Secondary metabolites biosynthesis, transport and catabolism	0	0	0	0.46
*Poorly characterized*	Function unknown	3.48	0	9.73	3.95
	General function prediction only	0	0	3.36	7.20

**Figure 9:**
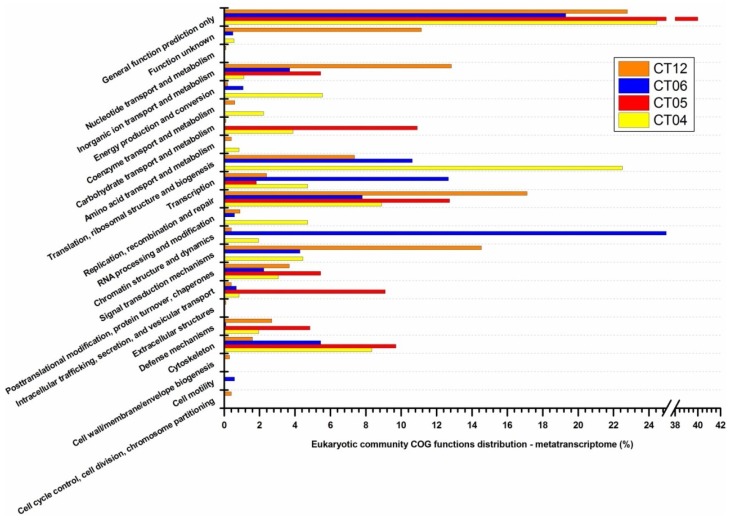
COG distributions of eukaryotic metatranscriptomic libraries.

The RNA/DNA ratio was again employed as an index to describe the metabolic strength of deep-sea eukaryotes. *Placozoa*, considered the simplest organized metazoan model system [75,76], showed the highest SMA value in all four deep-sea sampling sites (Figure 9). The highly active metabolism of *Placozoa* in the cold, dark, deep sea is quite surprising, because it has been suggested that the growth rate and vegetative reproduction of *Placozoa* may be positively correlated to increasing temperature [77]. Deep-sea autotrophic ecosystems, such as hydrothermal vents or cold seeps, are normally not considered conducive living environments for fungi that are abundant in terrestrial ecosystems because of their ability to degrade organic matter [78,79]. However, in our study the fungi *Basidiomycota* and *Microsporidia* showed the highest SMA value in site CT12, suggesting their affinity for the hydrothermal vent-influenced habitat. These results are consistent with the unexpected diversity of fungal species, such as *Basidiomycota*, detected in hydrothermal areas in previous studies [78,80]. 

## 3. Experimental Section

### 3.1. Microbial Sample Collection and DNA/RNA Isolation

Microbial samples for this study were collected from waters in four deep-sea Northeast Pacific Ocean sites during the Thompson TN 221 Research Cruise between 28 July 2008 and 2 August 2008, as described in Table 1. The more detail about sampling process has been described and published earlier [24,65]. Among these sampling sites, CT04, CT05 and CT06 are 164.2 km east of Newport, Oregon, while CT12 is much further away from the coast, lying approximately 20 km southwest of a deep-sea hydrothermal vent in the Juan de Fuca Ridge. At each site a total of 30 liters of deep-sea water was collected in six rigid, tightly-sealed 5-liter bottles carried on the Woods Hole Oceanographic Institute’s Towed Camera System (“TowCam”). The entire TowCam sampling excursion into the deep and return to the surface took about 1 h. Deep sea water was immediately filtered through 0.22-μm filters which were then stored at −80 °C till use. The filtration time for each single filter (sample) was about 0.5 h. To preserve the RNA profiles’ integrity, cells were gently washed from the filter membranes with chilled RNAlater solution (Ambion, Austin, TX, USA). A total of five 5.0-mL washes were performed for each filter, and cells recovered from the five washes were combined into a single pool and stored immediately at −80 °C for later DNA/RNA isolation. Genomic DNA was isolated with the DNeasy Blood and Tissue Kit (Qiagen, Valencia, CA, USA) following the bacterial DNA isolation protocol provided by the manufacturer. Total RNA was extracted from the collected microbial cells using a modified protocol combining Trizol (Invitrogen, Grand Island, NY, USA) and the prescribed RNeasy methods (QIAGEN, Valencia, CA, USA). The detailed procedures for sampling, storage and nucleic acid isolation have been described in previous papers [24,65].

### 3.2. DNA/RNA Amplification, Second Strand cDNA Synthesis, and Quality Examination

Genomic DNA and total RNA (rRNA and mRNA) were isolated, purified and amplified for pyrosequencing and the more detail has been described previously [24]. Briefly, the Ovation WGA system (NuGEN, San Carlos, CA, USA) was employed to amplify isolated DNA following the manufacturer’s protocol. After amplification, the products were purified with the QIAquick^®^ PCR Purification kit (QIAGEN, Valencia, CA, USA). The concentration and purity were determined using a NanoDrop (Thermo Scientific, West Palm Beach, FL, USA). Total RNA amplification was performed using the WT-Ovation™ Pico RNA Amplification System (NuGEN, San Carlos, CA, USA). The products from transcriptome amplification were purified and measured in the same way as DNA amplification products. DNA and RNA amplification products, both single-stranded, were converted into double-stranded products before conducting clone library construction and sequencing using the WT-Ovation™ Exon Module (NuGEN, CA, USA) method described previously [24]. To check the quality of amplification products and preclude the possibility of external contamination, we constructed clone libraries for the amplified products of each sample. The isolated plasmid DNA/cDNA from 20 random clones from each library were sequenced using ABI 373 Sequencer (Applied Biosystem Inc., Carlsbad, CA, USA) and BLAST annotated against the NCBI databases. 

### 3.3. Pyrosequencing

Approximately 5 µg of amplified DNA and double stranded cDNA from each sample were sequenced with Roche GS FLX Titanium chemistry pyrosequencing (Roche 454 Life Science, Branford, CT, USA), according to the established protocols provided by Engencore Inc. (University of South Carolina, Columbia, SC, USA). 

### 3.4. Data Analysis

We used the MG-RAST metagenomics analysis server provided by Argonne National Laboratory to perform phylogenetic and functional analysis on the metagenomic and metatranscriptomic data [49]. In MG-RAST version 3, reads are considered replicates if the first 50 bp are identical. De-replication was processed when uploading data. The M5NR, a searchable database integrated from several existing sequence databases, including KEGG (Kyoto Encyclopedia of Genes and Genomes), NCBI (National Center for Biotechnology Information), SEED (The SEED Project), and COG (Clusters of Orthologous Groups of proteins), was used to perform organism hierarchical classification and functional hierarchical classification searches in the pyrosequencing data. All searches were performed using the default parameters suggested by the MG-RAST server.

## 4. Conclusions

Microbial communities in seawater at four deep-sea sites were isolated and amplified to perform taxonomic and functional analyses by using an integrated metagenomic and metatranscriptomic approach. The results showed that within the prokaryote community bacteria is absolutely dominant over archaea (~90%) in both metagenomic and metatranscriptomic pools in the deep-sea prokaryotic samples. When compared with the microbial communities of the GOS surface water, the proportion of archaea in the prokaryotic community was increased in the deep-sea water. The emergence of archaeal phyla *Crenarchaeota*, *Euryarchaeota* and *Thaumarchaeota*, bacterial phyla *Actinobacteria*, *Firmicutes*, sub-phyla *Betaproteobacteria*, *Deltaproteobacteria*, and *Gammaproteobacteria*, and the decrease of bacterial phyla *Bacteroidetes* and *Alphaproteobacteria* were the main differences observed in the prokaryotic community compositions present in the deep-sea water. *Cyanobacteria* were identified in samples from all four deep-sea sites by metagenomic and metatranscriptomic analysis, suggesting their active functionality in deep-sea environments in spite of very little sunlight. Employing the RNA/DNA ratio as a metric indicative of the metabolic strength of microbes, we found that the metabolic strength of single phylum/sub-phylum varied remarkably across deep-sea sites, suggesting that the prokaryotic communities are experiencing distinctly different metabolic conditions at the different sites. In contrast to the GOS surface water communities, functional groups related to cell wall/membrane and capsule forming for high pressure resistance; signal sensing and transduction for adapting to the low-nutrient deep-sea environment; multidrug efflux for intrinsic and acquired resistance to antimicrobials; and defense mechanisms for self-protection were enhanced in the deep-sea water, indicative of a defensive life style rather than an active growing/metabolic style on the part of the prokaryotic community living in the deep sea. Taxonomic and functional analysis of the CT12 site, located 20 km away from the Juan de Fuca hydrothermal vent, harbored higher diversity than other deep-sea sites. In addition, decreases in abundance of fungi and algae in the deep sea were detected in our eukaryote study. Similar to prokaryotes, COG distribution analysis revealed that eukaryotes adapted a more defensive life style in the harsh deep-sea environments. This study provides the first integrated genomic and transcriptomic view of the microbial communities in deep-sea water of the North Pacific Ocean. It gave insight into deep-sea microbial community, the significant new source of drug discovery and development.

## Supplementary Files

Supplementary File 1 Supplementary Materials (PDF, 88 KB)
