# Supplementary material for: Integrated Metagenomic and Metatranscriptomic Analyses of Microbial Communities in the Meso- and Bathypelagic Realm of North Pacific Ocean
Source: Mar Drugs. 2013 Oct 11;11(10):3777–801. doi: 10.3390/md11103777 (PMC3826135; doi:10.3390/md11103777)
Supplement: Supplementary File 1 — Supplementary Materials (PDF, 88 KB) [file marinedrugs-11-03777-s001.pdf]

Supplementary Materials

Table S1. Cyanobacteria identified in metagenomic and metatranscriptomic.

Samples	Level 1	Function	Closest Species
CT04D	Amino Acids and Derivatives	Proline iminopeptidase (EC 3.4.11.5)	*Nostoc punctiforme* (strain ATCC 29133/PCC 73102)
	Carbohydrates	Gluconolactonase (EC 3.1.1.17)	*Synechococcus* sp. CC9311
		Acetate permease ActP (cation/acetate symporter)	*Anabaena* sp. PCC 7120 (*Nostoc* sp. PCC 7120)
		Mannose-1-phosphate guanylyltransferase (GDP) (EC 2.7.7.22)	*Synechococcus* sp. (strain WH7803)
	Clustering-based subsystems	Phytoene desaturase (EC 1.14.99.-)	*Nostoc punctiforme* (strain ATCC 29133/PCC 73102)
		Adenylate cyclase	*Anabaena* sp. PCC 7120 (*Nostoc* sp. PCC 7120)
	Cofactors, Vitamins, Prosthetic Groups, Pigments	5-amino-6-(5-phosphoribosylamino)uracil reductase (EC 1.1.1.193)	*Synechococcus elongatus* PCC 7942
		Diaminohydroxyphosphoribosylaminopyrimidine deaminase (EC 3.5.4.26)	*Synechococcus elongatus* PCC 7942
	Fatty Acids, Lipids, and Isoprenoids	Phytoene desaturase (EC 1.14.99.-)	*Nostoc punctiforme* (strain ATCC 29133/PCC 73102)
	Miscellaneous	Adenylate cyclase (EC 4.6.1.1)	*Anabaena* sp. PCC 7120 (*Nostoc* sp. PCC 7120)
		Serine peptidase (Alpha/beta hydrolase superfamily) fused to N- terminal uncharacterized domain specific to cyanobacteria	*Synechococcus* sp. CC9311
		Na+/H+ antiporter	*Nostoc punctiforme* PCC 73102
	Potassium metabolism	Potassium voltage-gated channel subfamily KQT	*Synechococcus* sp. RCC307
	Protein Metabolism	Acetate permease ActP (cation/acetate symporter)	*Anabaena* sp. PCC 7120 (*Nostoc* sp. PCC 7120)
		Selenide,water dikinase (EC 2.7.9.3)	*Microcystis aeruginosa* (strain NIES-843)
	Regulation and Cell signaling	Adenylate cyclase (EC 4.6.1.1)	*Anabaena* sp. PCC 7120 (*Nostoc* sp. PCC 7120)
	Respiration	ATP synthase alpha chain (EC 3.6.3.14)	*Crocosphaera watsonii* WH 8501
		Cytochrome c oxidase polypeptide I (EC 1.9.3.1)	*Prochlorococcus marinus* NATL1A
		soluble [2Fe-2S] ferredoxin	*Cyanothece* sp. (strain PCC 7425/ATCC 29141)
	Virulence, Disease and Defense	Negative regulator of beta-lactamase expression	*Synechococcus* sp. WH 7805
CT04R	Monosaccharides	Ribokinase (EC 2.7.1.15)	*Synechococcus* sp. RS9917
	Sugar utilization in Thermotogales	Ribokinase (EC 2.7.1.15)	*Synechococcus* sp. RS9917

Table S1. *Cont.*

CT05D	Amino Acids and Derivatives	5,10-methylenetetrahydrofolate reductase (EC 1.5.1.20)	*Synechococcus* sp. CC9605
	Carbohydrates	5,10-methylenetetrahydrofolate reductase (EC 1.5.1.20)	*Synechococcus* sp. CC9605
	Carbohydrates	decarboxylase	*Prochlorococcus marinus* MIT 9313
	Clustering-based subsystems	Adenylosuccinate synthetase (EC 6.3.4.4)	*Cyanothece* sp. PCC 7425
	Cofactors, Vitamins, Prosthetic Groups, Pigments	5,10-methylenetetrahydrofolate reductase (EC 1.5.1.20)	*Synechococcus* sp. CC9605
	Miscellaneous	Scaffold protein for [4Fe-4S] cluster assembly, MRP-like, similar to chloroplast-targeted plant protein HCF101	*Synechocystis* sp. (strain ATCC 27184/PCC 6803/N-1)
	Nucleosides and Nucleotides	Adenylosuccinate synthetase (EC 6.3.4.4)	*Cyanothece* sp. PCC 7425
	Photosynthesis	photosystem II protein D1 (PsbA)	*Synechococcus* sp. RCC307
	Protein Metabolism	Arginyl-tRNA synthetase (EC 6.1.1.19)	*Synechococcus* sp. CC9605
	Protein Metabolism	Chaperone protein HtpG	*Synechococcus* sp. (strain WH8102)
	Stress Response	Glutamate decarboxylase (EC 4.1.1.15)	*Prochlorococcus marinus* (strain MIT 9313)
CT06D	Amino Acids and Derivatives	Threonine dehydratase, catabolic (EC 4.3.1.19)	*Anabaena variabilis* (strain ATCC 29413/PCC 7937)
		Urease accessory protein UreG	Cyanobacteria bacterium Yellowstone B-Prime (*Synechococcus* sp. JA-2-3B'a(2-13))
		Threonine dehydratase, catabolic (EC 4.3.1.19)	*Anabaena variabilis* (strain ATCC 29413/PCC 7937)
		Glutamate synthase [NADPH] small chain (EC 1.4.1.13)	*Cyanothece* sp. PCC 7424
		Threonine dehydratase, catabolic (EC 4.3.1.19)	*Anabaena variabilis* (strain ATCC 29413/PCC 7937)
	Carbohydrates	Transketolase (EC 2.2.1.1)	*Nostoc punctiforme* PCC 73102
		L-alanine:glyoxylate aminotransferase (EC 2.6.1.44)	*Cyanothece* sp. (strain PCC 7424) (*Synechococcus* sp. (strain ATCC 29155))
		Transketolase (EC 2.2.1.1)	*Nostoc punctiforme* PCC 73102
		Mannose-1-phosphate guanylyltransferase (GDP) (EC 2.7.7.22)	*Synechococcus* sp. CC9902
		Serine--pyruvate aminotransferase (EC 2.6.1.51)	*Cyanothece* sp. (strain PCC 7424) (*Synechococcus* sp. (strain ATCC 29155))
		Transketolase (EC 2.2.1.1)	*Nostoc punctiforme* PCC 73102

Table S1. *Cont.*

	Cell Division and Cell Cycle	Carbamoyl-phosphate synthase large chain (EC 6.3.5.5)	*Prochlorococcus marinus* MIT 9303
	Cell Wall and Capsule	Mannose-1-phosphate guanylyltransferase (GDP) (EC 2.7.7.22)	*Synechococcus* sp. CC9902
	Clustering-based subsystems	Peptidyl-tRNA hydrolase (EC 3.1.1.29)	*Synechococcus elongatus* PCC 7942
	Cofactors, Vitamins, Prosthetic Groups, Pigments	Molybdenum cofactor biosynthesis protein MoaD<br>Glutamate synthase [NADPH] small chain (EC 1.4.1.13)<br>Flavodoxin<br>Flavodoxin 1<br>Sulfur carrier protein adenylyltransferase ThiF	*Thermosynechococcus elongatus* (strain BP-1)<br>*Cyanothece* sp. PCC 7424<br>*Synechococcus* sp. CC9311<br>Synechococcus sp. CC9311<br>*Cyanobacteria bacterium* Yellowstone B-Prime (*Synechococcus* sp. JA-2-3B'a(2-13))
	Dormancy and Sporulation	Peptidyl-tRNA hydrolase (EC 3.1.1.29)	*Synechococcus elongatus* PCC 7942
	Membrane Transport	Dipeptide transport ATP-binding protein DppD (TC 3.A.1.5.2)	*Cyanothece* sp. ATCC 51142
	Nitrogen Metabolism	Glutamate synthase [NADPH] small chain (EC 1.4.1.13)	*Cyanothece* sp. PCC 7424
	Nucleosides and Nucleotides	Carbamoyl-phosphate synthase large chain (EC 6.3.5.5)	*Prochlorococcus marinus* MIT 9303
	Photosynthesis	Octaprenyl-diphosphate synthase (EC 2.5.1.-)	-
	Protein Metabolism	SSU ribosomal protein S6p<br>Peptidyl-tRNA hydrolase (EC 3.1.1.29)<br>Prolyl-tRNA synthetase (EC 6.1.1.15)<br>Chaperone protein DnaJ<br>Urease accessory protein UreG	*Prochlorococcus marinus* SS120 (subsp. *marinus* CCMP1375)<br>*Synechococcus elongatus* PCC 7942<br>*Synechococcus* sp. CC9902<br>*Synechococcus* sp. WH 7803<br>*Thermosynechococcus elongatus* BP-1<br>*Cyanobacteria bacterium* Yellowstone B-Prime (*Synechococcus* sp. JA-2-3B'a(2-13))
	Stress Response	Chaperone protein DnaJ<br>5-oxoprolinase (EC 3.5.2.9)	*Synechococcus* sp. WH 7803<br>*Cyanothece* sp. (strain ATCC 51142)
CT06R	Photosynthesis	photosystem II protein D1 (PsbA)	*Nostoc* sp. (strain PCC 7120 / UTEX 2576)

Table S1. *Cont.*

CT12D	Amino Acids and Derivatives	Glutamate *N*-acetyltransferase (EC 2.3.1.35)	*Synechococcus* sp. JA-2-3B'a(2-13)
		N-acetylglutamate synthase (EC 2.3.1.1)	*Synechococcus* sp. JA-2-3B'a(2-13)
	Carbohydrates	Succinate dehydrogenase flavoprotein subunit (EC 1.3.99.1)	*Anabaena* sp. PCC 7120 (*Nostoc* sp. PCC 7120)
	Cell Wall and Capsule	Bacillosamine/Legionaminic acid biosynthesis aminotransferase PglE	*Prochlorococcus marinus* (strain MIT 9303)
		ADP-heptose synthase (EC 2.7.-.-)	*Cyanothece* sp. (strain PCC 7424) (*Synechococcus* sp. (strain ATCC 29155))
		D-glycero-beta-D-manno-heptose 7-phosphate kinase	*Cyanothece* sp. (strain PCC 7424) (*Synechococcus* sp. (strain ATCC 29155))
	Clustering-based subsystems	Ribosomal-protein-S18p-alanine acetyltransferase (EC 2.3.1.-)	*Microcystis aeruginosa* NIES-843
		Glycosyltransferase	*Anabaena variabilis* ATCC 29413
		Glycosyltransferase (EC 2.4.1.-)	*Anabaena variabilis* ATCC 29413
		Translation elongation factor Ts	*Prochlorococcus marinus* (strain MIT 9303)
		Alanyl-tRNA synthetase (EC 6.1.1.7)	*Anabaena variabilis* ATCC 29413
		Amino acid permease in hypothetical Actinobacterial gene cluster	*Cyanothece* sp. (strain PCC 7425/ATCC 29141)
		Helicase PriA essential for oriC/DnaA-independent DNA replication	*Cyanothece* sp. PCC 7425
		Ribosomal-protein-S18p-alanine acetyltransferase (EC 2.3.1.-)	*Microcystis aeruginosa* NIES-843
		Competence/damage-inducible protein CinA	*Synechococcus* sp. (strain JA-3-3Ab) (*Cyanobacteria bacterium* Yellowstone A-Prime)
	Cofactors, Vitamins, Prosthetic Groups, Pigments	Phosphomethylpyrimidine kinase (EC 2.7.4.7)	*Prochlorococcus marinus* (strain MIT 9215)
		Orotate phosphoribosyltransferase (EC 2.4.2.10)	*Synechococcus* sp. (strain RCC307)
		Phosphomethylpyrimidine kinase (EC 2.7.4.7)	*Prochlorococcus marinus* (strain MIT 9215)
		Sulfur carrier protein adenylyltransferase ThiF	*Cyanothece* sp. PCC 8801
	DNA Metabolism	Helicase PriA essential for oriC/DnaA-independent DNA replication	*Cyanothece* sp. PCC 7425

Table S1. *Cont.*

Miscellaneous	Membrane protein PxcA, involved in light-induced proton extrusion	*Synechocystis* sp. (strain ATCC 27184/PCC 6803/N-1)27184/ PCC 6803/N-1)
	Glutathione *S*-transferase family protein	*Prochlorococcus marinus* (strain MIT 9313)
	Ribosomal-protein-S18p-alanine acetyltransferase (EC 2.3.1.-)	*Microcystis aeruginosa* NIES-843
	Glycosyltransferase	*Anabaena variabilis* ATCC 29413
	Phosphomethylpyrimidine kinase (EC 2.7.4.7)	*Prochlorococcus marinus* (strain MIT 9215)
	Competence/damage-inducible protein CinA	*Synechococcus* sp. (strain JA-3-3Ab) (*Cyanobacteria bacterium* Yellowstone A-Prime)
Nucleosides and Nucleotides	Orotate phosphoribosyltransferase (EC 2.4.2.10)	*Synechococcus* sp. (strain RCC307)
Phages, Prophages, Transposable elements, Plasmids	Heat shock protein 60 family chaperone GroEL	*Prochlorococcus marinus* MED4 (subsp. pastoris str. CCMP1378)
Photosynthesis	photosystem II protein D1 (PsbA)	*Cyanothece* sp. (strain PCC 8802) (*Synechococcus* sp. (strain RF-2))
Protein Metabolism	Translation elongation factor Ts	*Prochlorococcus marinus* (strain MIT 9303)
	Translation elongation factor Tu	*Cyanobacteria bacterium* Yellowstone A-Prime (*Synechococcus* sp. JA-3-3Ab)
	Alanyl-tRNA synthetase (EC 6.1.1.7)	*Anabaena variabilis* ATCC 29413
	Valyl-tRNA synthetase (EC 6.1.1.9)	*Trichodesmium erythraeum* (strain IMS101)
	Prolyl endopeptidase (EC 3.4.21.26)	*Gloeobacter violaceus* PCC7421
	Heat shock protein 60 family chaperone GroEL	*Prochlorococcus marinus* MED4 (subsp. pastoris str. CCMP1378)
	4-keto-6-deoxy-*N*-Acetyl-D-hexosaminyl-(Lipid carrier) aminotransferase	*Prochlorococcus marinus* (strain MIT 9303)
Respiration	Succinate dehydrogenase flavoprotein subunit (EC 1.3.99.1)	*Anabaena* sp. PCC 7120 (*Nostoc* sp. PCC 7120)
Stress Response	Glutathione *S*-transferase family protein	*Prochlorococcus marinus* (strain MIT 9313)
Carbohydrates	Ribokinase (EC 2.7.1.15)	*Synechococcus* sp. RS9917
Fatty Acids, Lipids, and Isoprenoids	Phytoene desaturase, pro-zeta-carotene producing (EC 1.-.-.-)	*Synechococcus elongatus* (strain PCC 7942) (*Anacystis nidulans* R2)
Phosphorus Metabolism	Alkaline phosphatase (EC 3.1.3.1)	*Cyanothece* sp. CCY0110
